# Cholesterol 25-hydroxylase inhibits Newcastle disease virus replication by enzyme activity-dependent and direct interaction with nucleocapsid protein

**DOI:** 10.1128/jvi.00428-25

**Published:** 2025-04-22

**Authors:** Guangmei Zhu, Xianchun Zong, Mengmeng Xiao, Dan Wang, Zhe Xu, Jingtao Hu, Guilian Yang, Yanlong Jiang, Wentao Yang, Haibin Huang, Chunwei Shi, Yan Zeng, Nan Wang, Xin Cao, Jianzhong Wang, Chunfeng Wang

**Affiliations:** 1 College of Veterinary Medicine, Jilin Agricultural University 85112, Changchun, China; 2 Jilin Provincial Engineering Research Center of Animal Probiotics, Jilin Agricultural University 85112, Changchun, China; 3 Engineering Research Center of Microecological Vaccines (Drugs) for Major Animal Diseases, Ministry of Education, Jilin Agricultural University 85112, Changchun, China; Loyola University Chicago - Health Sciences Campus, Maywood, Illinois, USA

**Keywords:** Newcastle disease virus, cholesterol 25-hydroxylase, 25-hydroxycholesterol, viral replication

## Abstract

Newcastle disease virus (NDV) is a significant enveloped virus within the Paramyxoviridae family, posing a major threat to the global poultry industry. Increasing evidence suggests that cholesterol-25-hydroxylase (CH25H) and its enzymatic product, 25-hydroxycholesterol (25HC), exhibit broad-spectrum antiviral activity properties by modulating lipid metabolism and various signaling pathways. However, the specific role of CH25H in regulating NDV infection and replication remains unclear. In this study, we demonstrate that CH25H significantly inhibits NDV replication by blocking viral entry through its enzymatic product, 25HC. Notably, a catalytic mutant of CH25H (CH25H-M), which lacks hydroxylase activity, still retains partial ability to inhibit NDV replication, suggesting the involvement of an enzyme-independent antiviral mechanism. Furthermore, we found that CH25H interacts with the viral nucleoprotein (NP), leading to a reduction in NP expression and inhibition of viral ribonucleoprotein (RNP) complex activity. These findings reveal that CH25H exerts antiviral effects through both enzyme-dependent and -independent mechanisms, providing new insights into its role in host defense and offering potential targets for the development of antiviral therapies.

**IMPORTANCE:**

Cholesterol 25-hydroxylase (CH25H) is a multifunctional host protein that has been implicated in regulating the life cycles of various viruses. As a prototype of paramyxovirus, Newcastle disease virus (NDV) poses a significant threat to the global poultry industry, causing substantial economic losses. Uncovering the mechanisms of NDV-host interactions is crucial for unraveling the viral pathogenesis and the host immune response, which can drive the development of effective antiviral therapies. Here, we demonstrate that CH25H, whose expression is induced upon NDV infection, plays a pivotal role in restricting viral replication. Specifically, CH25H interacts with the viral NP and inhibits the viral RNP activity. These findings expand our understanding of CH25H’s antiviral functions and offer new insights into virus-host interactions, providing potential targets for the development of novel antiviral drugs against NDV and related pathogens.

## INTRODUCTION

Paramyxoviruses represent a large family of enveloped RNA viruses that include highly infectious pathogens that affect both humans and animals, leading to significant health and economic burdens worldwide (1). In poultry, the most important paramyxovirus, Newcastle disease virus (NDV), also known as *Avian paramyxovirus-1* (APMV-1), *Avian avulavirus 1* (AAvV-1), recently renamed *Avian orthoavulavirus 1* (AOAV-1), belongs to genus *Orthoavulavirus* within the family of *Paramyxoviridae* (2). NDV is infective for almost all species of birds and a leading cause of economic losses to the global poultry industry. Similar to other paramyxoviruses, the virus also infects non‐avian hosts, including *Bovidae* (cattle and sheep), *Mustelidae* (minks), *Cercetidae* (hamsters), *Muridae* (mice), *Leporidae* (rabbits), *Camelidae* (camels), *Suidae* (pigs), *Cercophithecidae* (monkeys), and *Hominidae* (humans), potentially in a lethal form in immunocompromised humans, as well as in minks, pigs, and cattle (3). The genome of NDV is a linear, non-segment, negative-sense single-stranded RNA that encodes nucleocapsid (N) protein, phosphoprotein (P), matrix protein (M), fusion protein (F), hemagglutinin-neuraminidase (HN), and large polymerase (L). Additionally, it encodes two nonstructural proteins, V and W. Among these proteins, nucleoprotein (NP) is highly expressed in NDV and primarily regulates the replication of its genomic RNA, playing a crucial role in the replication process (4).

Upon infection, NDV accomplishes its lifecycle through the stages of attachment, fusion, uncoating, replication, assembly, and budding (5). Cellular lipids, including cholesterol and its derivatives, participate in different steps of replication. Suppressing cholesterol metabolism or reducing cellular cholesterol can reduce viral entry and replication (6). Cholesterol-25-hydroxylase (CH25H), an endoplasmic-reticulum-associated enzyme that catalyzes the oxidation of cholesterol to 25-hydroxycholesterol (25HC), is involved in cholesterol and lipid metabolism. CH25H has been identified as a conserved interferon-stimulated gene (ISG) induced upon viral detection, playing a key role in the innate immune response across mammalian species (7), chickens (8), and fish (9, 10). Accumulating evidence suggests that CH25H and its enzymatic product 25HC exhibit broad-spectrum antiviral activity and inhibit the proliferation of multiple viruses by regulating lipid metabolism and signaling pathways (11). For some viruses, the antiviral effect of CH25H strictly depends on its enzymatic activity. A catalytic mutant CH25H, which loses cholesterol 25-hydroxylase activity, failed to inhibit Hantavirus (12), Avian reovirus (13), Seneca Valley virus (14), VSV-SARS-CoV-2 chimeric virus (15), and Rabies virus (RABV) infection. However, both CH25H and its mutants still inhibit the replication of Porcine deltacoronavirus(16), Classical swine fever virus (17), Encephalomyocarditis virus (18), Bovine Parainfluenza Virus Type 3 (19), and Porcine epidemic diarrhea virus (20).

As a natural host restriction factor, CH25H can also directly interact with viral coding proteins to inhibit viral replication, demonstrating its diverse antiviral mechanisms. Studies have shown that human CH25H interacts with nonstructural protein 5A (NS5A) (21) and the C-terminal region of hepatitis B virus (HBV)-encoded X protein (HBx) (22) to suppress the replication of hepatitis C virus and HBV, respectively. Similarly, porcine CH25H binds to and degrades the nonstructural protein 1 alpha (nsp1α) of Porcine reproductive and respiratory syndrome virus (PRRSV) through the ubiquitin-proteasome pathway, thereby restricting its replication (23). CH25H is suggested to hold potential for novel therapeutic approaches and the development of antiviral strategies. However, the antiviral effects and mechanisms of CH25H and 25HC on NDV remain unclear.

The present study aimed to investigate the expression and antiviral activity of chicken CH25H during NDV infection. Our finding revealed that CH25H significantly inhibits NDV infection by blocking viral entry in a manner dependent on its oxidation product, 25HC. Interestingly, a catalytic mutant of CH25H, CH25H-M, still retained the ability to inhibit NDV replication. Moreover, we discovered that CH25H interacts with and degrades the viral NP, further restricting NDV infection. These findings provide new evidence that CH25H inhibits NDV replication through both enzyme activity-dependent and -independent mechanisms, offering promising insights for the development of novel antiviral therapies against this virus.

## RESULTS

### The expression of CH25H gene is affected during NDV infection, and overexpressed CH25H inhibited replication of NDV

**Fig 1:**
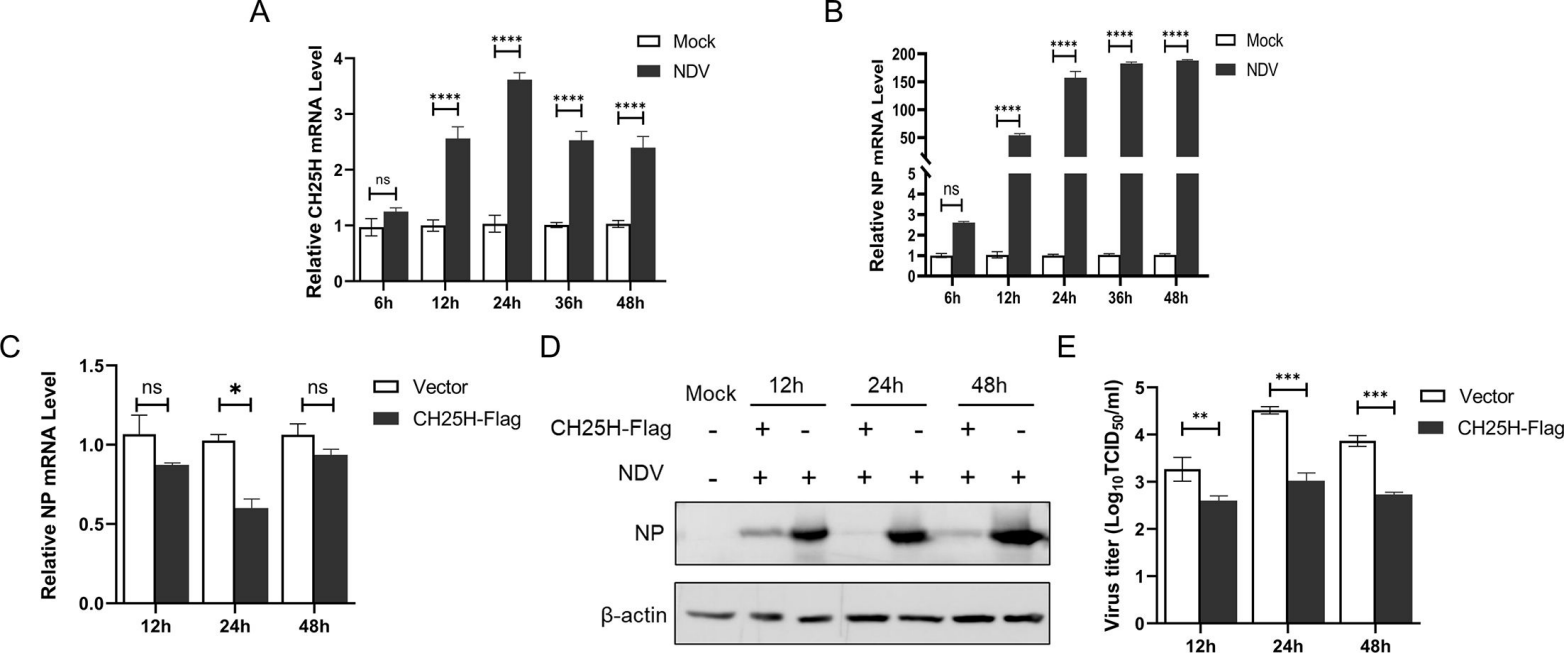
The CH25H was upregulated by NDV infection in DF-1 cells, and overexpressed CH25H inhibited replication of NDV. DF-1 cells were infected with NDV NA-1 strain at an MOI of 0.1. The mRNA levels of CH25H (**A**) and NP (**B**) were detected by qRT-PCR at 6, 12, 24, 36, and 48 hpi. DF-1 cells were transfected with either the Flag-tagged CH25H expressing plasmid or the empty vector pCAGGS for 24 h, followed by infection with NDV NA-1 at an MOI of 0.1. Cell samples and supernatants were harvested at 12, 24, and 48 hpi. NDV NP and mRNA levels were evaluated by qRT-PCR (**C**) and western blot analysis (**D**). The viral titers were quantified by TCID_50_ analysis (**E**). Results are presented as the mean ± SD from three independent experiments. *P < 0.05, **P < 0.01, ***P < 0.0001, ns (not significant), *P* > 0.05.

To analyze the expression of CH25H in NDV-infected DF-1 cells, DF-1 cells were infected with the NDV NA-1 strain and incubated for 6, 12, 24, 36, and 48 h at 37°C. The mRNA levels of CH25H and NP at different times were measured using qRT-PCR assays. The results showed that CH25H mRNA was dramatically upregulated starting at 12 hours post-infection (hpi), peaked at 24 hpi, and decreased at 36 hpi (Fig. 1A), while NDV NP mRNA levels were significantly increased with time (Fig. 1B).

To examine the effect of CH25H on NDV replication, DF-1 cells were transfected with either a CH25H-expressing plasmid or the empty pCAGGS vector. After 24 hpt, the cells were infected with NDV at an MOI of 0.1. Cell samples were collected at 12, 24, and 48 hpi, and NDV replication was evaluated by qRT-PCR, western blot analysis, and virus titration. Compared to the control group, NP mRNA levels were significantly reduced at 24 hpi and did not change notably at 12 and 48 hpi. However, both NDV NP levels and virus titers significantly reduced at 12, 24, and 48 hpi (Fig. 1C and D). These results demonstrated that overexpression of CH25H significantly suppressed the replication of NDV.

### Knockdown of CH25H by small interfering RNAs (siRNAs) enhances NDV replication

**Fig 2:**
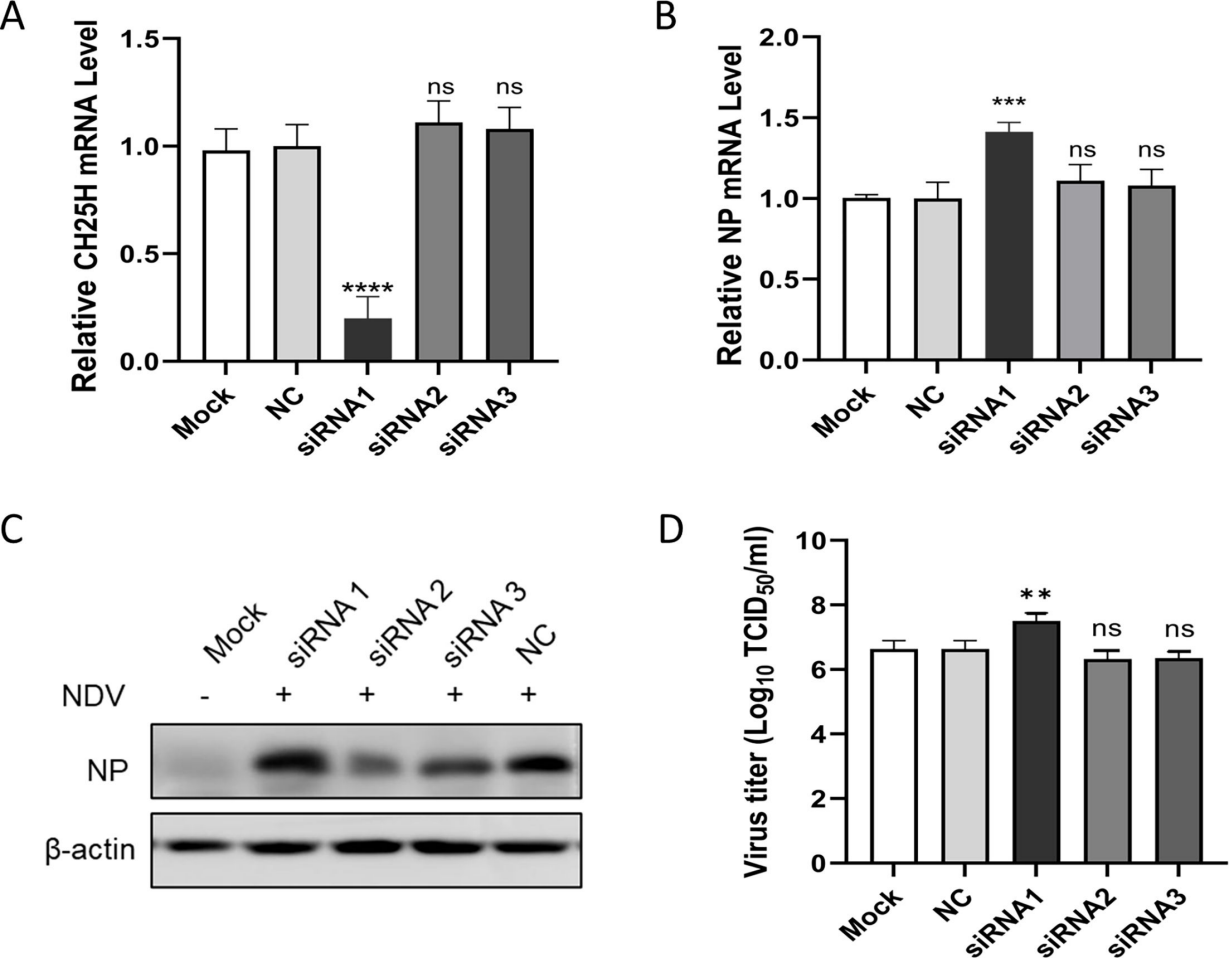
Knockdown of CH25H by siRNAs enhances NDV replication. DF-1 cells were transfected with three siRNAs (siRNA1, siRNA2, and siRNA3) or negative control (NC). At 48 hpt, the knockdown efficiency of CH25H was determined by qRT-PCR (**A**). DF-1 cells transfected with the three siRNAs were subsequently infected with the NDV NA-1 strain (MOI = 0.1) at 48 hpt. After 24 h of infection, cell samples were collected to assess the replication of NDV by qRT-PCR (**B**), western blot assays (**C**). Supernatants were collected for viral titer measurement by TCID_50_ analysis (**D**). Results are presented as mean ± SD from three independent experiments. *P < 0.05, **P < 0.01, ***P < 0.0001, ns (not significant), *P* > 0.05.

To further explore the antiviral effect of CH25H on NDV replication, DF-1 cells were transfected with three siRNAs targeting the CH25H gene. After 48 h, CH25H mRNA levels were detected by qRT-PCR. The results showed that the three siRNAs exhibited varied knockdown effects on CH25H expression, with siRNA1 displaying the highest knockdown efficiency (Fig. 2A). In DF-1 cells transfected with these siRNAs and subsequently infected by NDV infection (MOI = 0.1), NP mRNA levels and protein expression were increased in response to the downregulation of CH25H, compared to cells transfected with the negative control (NC) (Fig. 2B and C). The TCID_50_ assays also demonstrated that knockdown of CH25H significantly increased viral titers (Fig. 2D).

### Construction and characterization of recombinant NDV expressing CH25H

**Fig 3:**
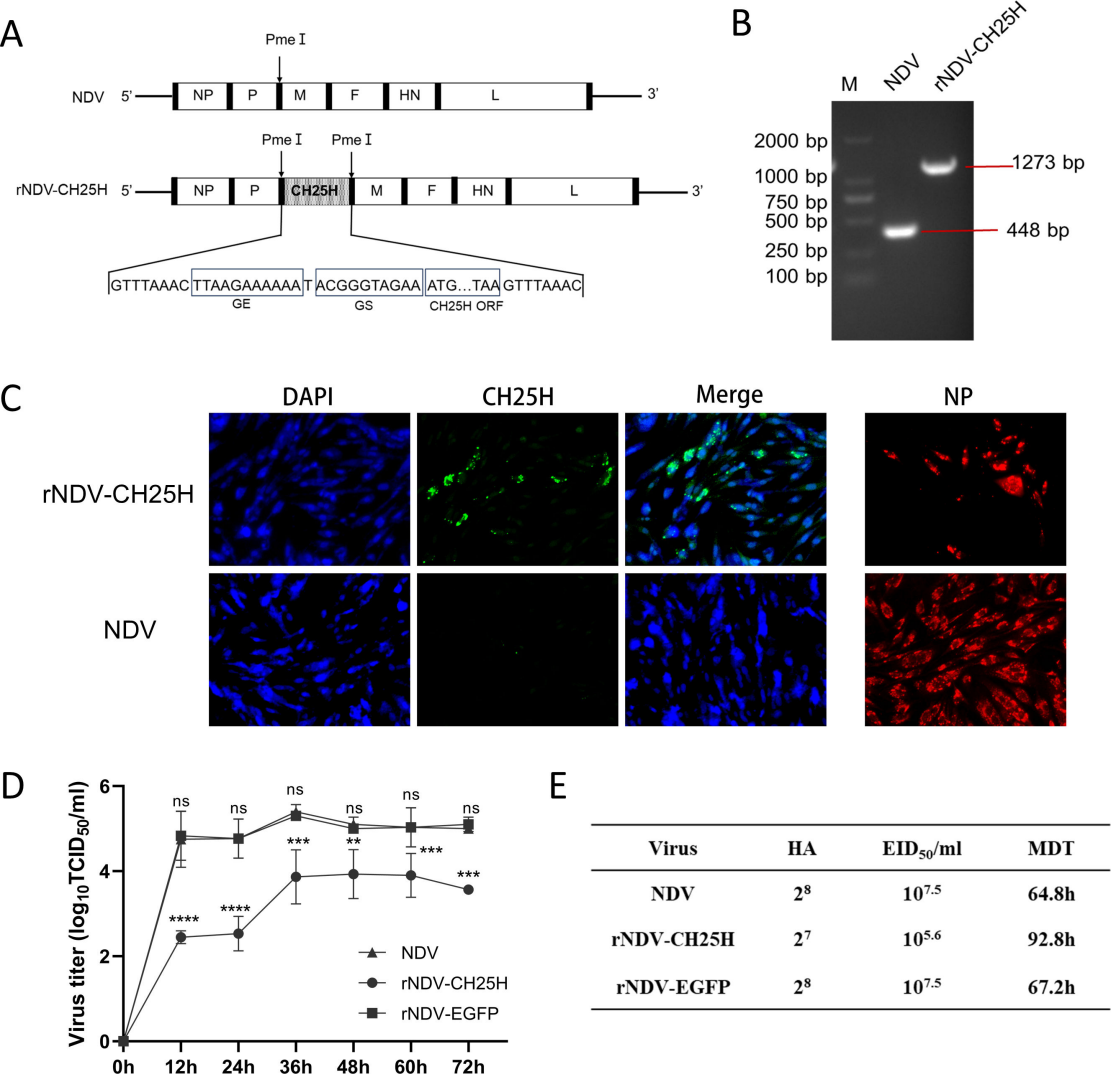
Construction and characterization of recombinant NDV expressing CH25H. (**A**) A schematic diagram illustrating the construction of recombinant NDV expressing CH25H. An artificial transcription cassette coding CH25H was inserted into the NDV genome between the P and M genes. (**B**) The insertion of the CH25H gene into rNDV-CH25H was verified by RT-PCR using a pair of primers spanning the insertion region of the CH25H gene. (**C**) DF-1 cells were infected with rNDV-CH25H at an MOI of 1. At 24 h post-infection, the cells were fixed and subjected to immunofluorescence assays to detect CH25H protein and NP. Primary antibodies (mouse anti-CH25H and rabbit anti-NP) were used, followed by secondary antibodies conjugated with AF488 (anti-mouse) and AF555 (anti-rabbit). (**D**) Multistep growth kinetics of the recombinant virus in DF-1 cells were analyzed. Cell monolayers were infected with rNDV-CH25H, rNDV-EGFP, or NDV NA-1 strain at an MOI of 1. Viruses were harvested at 12 h intervals, and virus titers were measured in DF-1 cells and expressed as TCID_50_/mL. (**E**) Biological characteristics of the recombinant viruses were evaluated. Data represent the mean values from triplicate experiments. *P < 0.05, **P < 0.01, ***P < 0.0001, ns (not significant, *P* > 0.05).

To further analyze the function of CH25H in restricting NDV, a recombinant NDV expressing CH25H was constructed based on reverse genetics with an artificial transcription cassette coding for CH25H inserted into the genome of NDV NA-1 strain between the genes P and M (Fig. 3A). The presence of the inserted CH25H gene in rNDV-CH25H was confirmed by RT-PCR (Fig. 3B) and sequencing. The expression of the CH25H or NP in DF-1 cells infected with rNDV and rNDV-CH25H at 24 h was identified by immunofluorescence staining. The expression of both the CH25H protein and NP was detected in rNDV-CH25H-infected cells, while only the NPs were detected in the NDV-infected cells (Fig. 3C).

To investigate the effect of CH25H on the replication of rNDV-CH25H, we analyzed the viral growth kinetics of rNDV-CH25H, rNDV-EGFP, and the parent NDV NA-1 strain in DF-1 cells. The results showed that rNDV-EGFP exhibited similar growth to the parent NDV NA-1 strain, while rNDV-CH25H displayed significantly reduced replication. The lowest infectious viral titer was observed in rNDV-CH25H-infected cells compared to those infected with rNDV-EGFP or the parent NDV strain (Fig. 3D), indicating that CH25H restricts NDV replication in DF-1 cells rather than the insertion of a foreign gene. Similarly, in chicken embryos, rNDV-CH25H exhibited a lower HA titer of 2^7^ and an EID_50_ of 10^5.6^ /mL, whereas rNDV-EGFP and the parental NDV both had an HA titer of 2^8^ and an EID_50_ of 10^7.5^ /mL. Additionally, the MDT of rNDV-CH25H significantly extended to 92.8 h, compared to 67.2 h for rNDV-EGFP and 64.8 h for the parental NDV NA-1 strain, demonstrating that rNDV-CH25H exhibited substantially reduced pathogenicity (Fig. 3E).

### 25HC inhibits NDV replication

**Fig 4:**
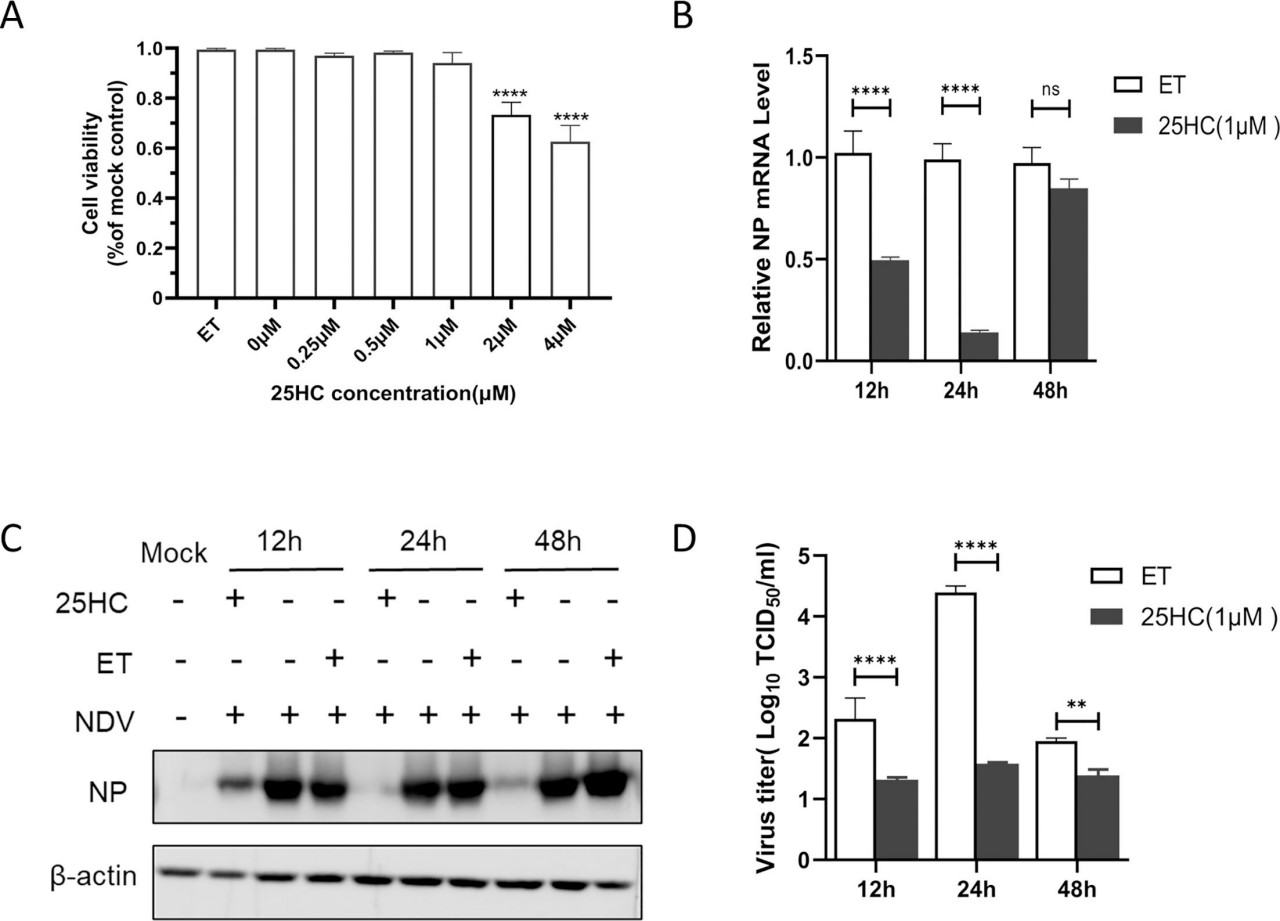
25HC inhibits NDV replication. DF-1 cells were treated with different concentrations of 25HC (0.25, 0.5, 1, 2, and 4 µM) or ethanol (ET) as a control for 24 h, and cell viability was assessed using the CCK-8 assay (**A**). To evaluate the effect of 25HC on NDV replication, DF-1 cells were pretreated with 1 µM 25HC for 24 h, and then infected with the NDV NA-1 strain at an MOI of 0.1. At 12, 24, and 48 hpi, cell samples and supernatants were collected and analyzed via qRT-PCR (**B**), western blot analysis (**C**), and TCID_50_ assay (**D**).

CH25H is an enzyme that catalyzes the oxidation of cholesterol to produce 25HC. To determine whether the antiviral effect of CH25H on NDV is dependent on its enzymatic activity, we first assessed the cytotoxicity of 25HC in DF-1 cells using the CCK-8 assay. The results showed that treatment with 25HC at concentrations of below 1 µM for 24 h exhibited no cytotoxic effect on DF-1 cells (Fig. 4A). Next, DF-1 cells were pretreated with 1 µM 25HC or ethanol (ET) as a control for 24 h, followed by infection with the NDV NA-1 strain at an MOI of 0.1. Viral replication was analyzed at 12, 24, and 48 hpi using qRT-PCR, western blot analysis, and TCID_50_ assays. Compared to the control, 25HC significantly reduced the mRNA levels of the viral NP gene at 12 and 24 hpi (Fig. 4B). Similarly, NP levels were notably reduced at these time points (Fig. 4C). Additionally, viral titers in the supernatants from NDV-infected DF-1 cells were markedly decreased (Fig. 4D). These findings demonstrate that 25HC effectively inhibits NDV replication in DF-1 cells, suggesting that the enzymatic product of CH25H, 25HC, plays a critical role in mediating this antiviral effect.

### 25HC inhibits NDV infection by blocking viral entry

**Fig 5:**
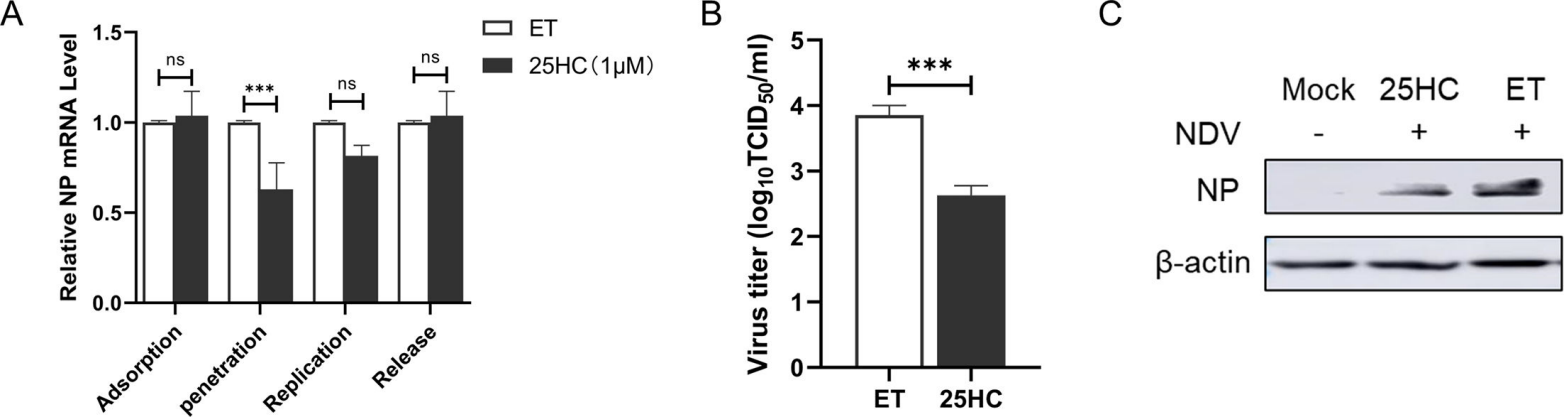
25HC blocks NDV penetration. For the attachment assay, DF-1 cells were pretreated with 1 µM 25HC or ET for 24 h at 37°C, then pre-chilled at 4°C for 1 h before infection with the NDV NA-1 strain (MOI = 10) for 2 h at 4°C. Cells were collected, and NDV NP mRNA levels were measured via qRT-PCR. For the penetration assay, DF-1 cells were pretreated with 25HC (1 µM) for 24 h, prechilled at 4°C for 1 h, then infected with NDV NA-1 strain (MOI = 10). After washing with PBS, the medium was replaced with DMEM containing 1 µM 25HC or ET, and cells were inoculated at 37°C for 2 h. Following three washes with PBS, cells were collected for qRT-PCR analysis of NP mRNA levels. For the replication assay, DF-1 cells were pretreated with 25HC (1 µM) at 37°C for 12 h, followed by NDV infection at 37°C. The infected cells were collected for qRT-PCR at 8 hpi. For the release assay, DF-1 cells were infected with NDV (MOI = 10). At 8 hpi, the virus-containing medium was replaced with fresh medium containing 1 µM 25HC or ET. After 2 h of 25HC treatment, cells were collected for qRT-PCR analysis (**A**). Supernatants and cell samples from the penetration assay were further analyzed via TCID_50_ (**B**) and western blot analysis (**C**). All results are presented as mean ± SD from three independent experiments, each performed in triplicate. *P < 0.05, **P < 0.01, ***P < 0.0001, ns: *P* > 0.05.

To further determine which stage of NDV replication is inhibited by 25HC, we examined the effects of 25HC on NDV during the attachment, penetration, replication, and release stages by quantifying viral NP mRNA levels. The results showed that NP mRNA levels in 25HC-treated cells were comparable to those in ET control cells during the attachment, replication, and release stages (Fig. 5A), suggesting that 25HC does not affect these phases of NDV infection. In contrast, at the penetration stage, 25HC treatment significantly reduced NP mRNA levels compared to the ET group. Additionally, NP expression and viral titers were both substantially downregulated (Fig. 5B and C), further confirming these findings. These results indicate that 25HC specifically inhibits NDV infection at the penetration stage.

### CH25H mutant lacking hydroxylase activity still inhibits NDV replication

**Fig 6:**
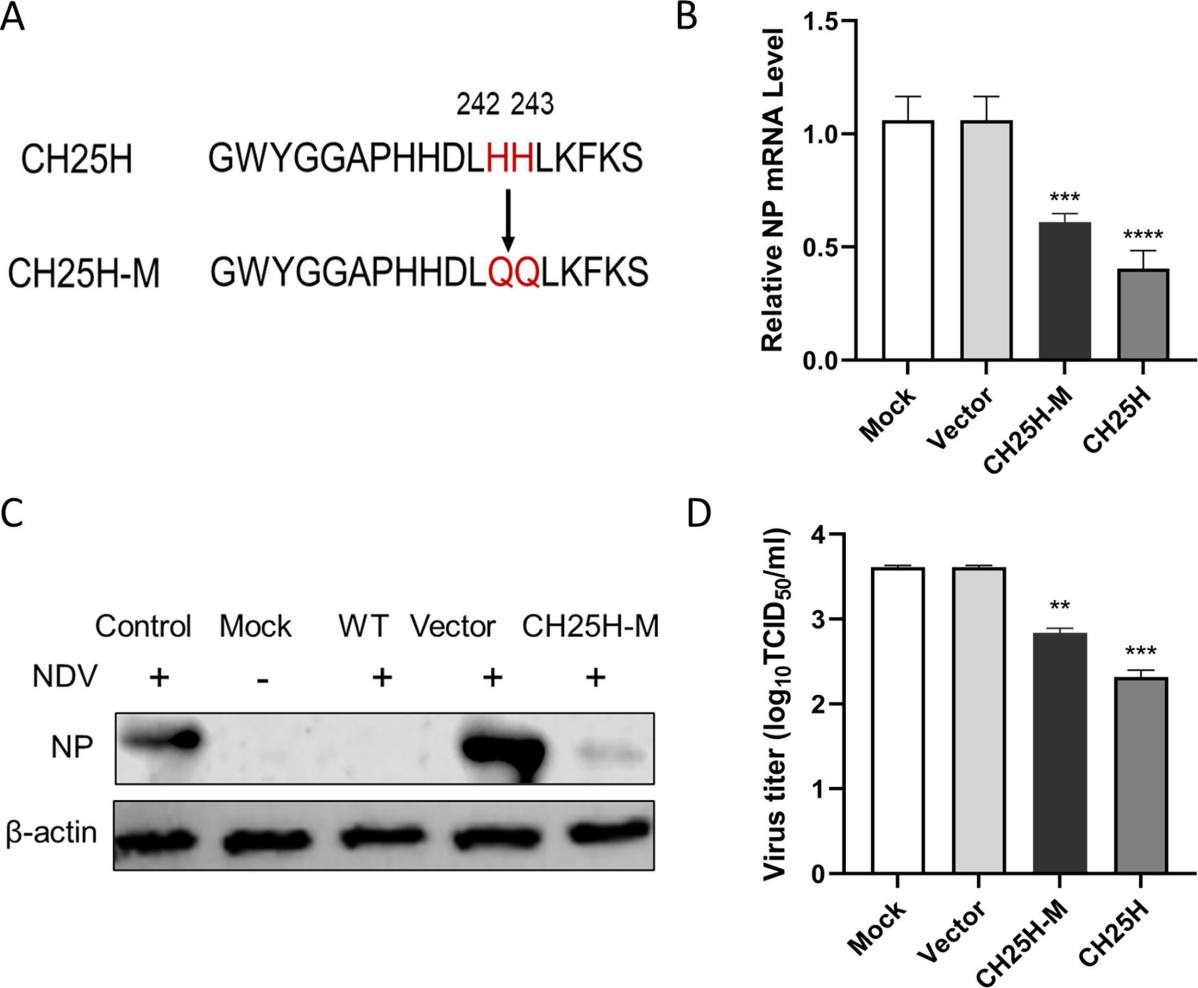
CH25H mutant lacking hydroxylase activity can suppress NDV replication. A catalytic mutant of CH25H (CH25H-M) was generated by substituting histidine residues at positions 242 and 243 with glutamine via site-directed mutagenesis (**A**). DF-1 cells were transfected with pCAGGS-CH25H, pCAGGS-CH25H-M, or the pCAGGS vector, then infected with the NDV NA-1 strain at an MOI of 0.1. After 24 h, cell samples and supernatants were collected for analysis by qRT-PCR (**B**), western blot analysis (**C**), and TCID_50_ assay (**D**). Data represent means ± SD from three independent experiments conducted in triplicate. *P < 0.05, **P < 0.01, ***P < 0.0001.

To investigate whether the enzymatic activity of CH25H is essential for its antiviral activity against NDV, we constructed a catalytic mutant of CH25H (CH25H-M) by substituting the conserved histidine residues 242 and 243 with glutamine, rendering it incapable of producing 25HC (Fig. 6A). DF-1 cells were transfected with plasmids expressing CH25H-M or wild-type CH25H, and then infected with NDV at an MOI of 0.1 at 24 h post-transfection. After 12 h of incubation, cell samples and supernatants were collected for qRT-PCR, western blot analysis, and TCID_50_ assays. The results showed that both CH25H-M and CH25H significantly downregulated NP mRNA levels and expression levels compared to the vector control (Fig. 6B and C). In addition, a marked reduction in viral titers was observed in both the CH25H-M and CH25H groups (Fig. 6D). These data demonstrate that CH25H-M lacking hydroxylase activity retains its inhibitory effect on NDV replication.

### CH25H interacts with NP and downregulates its expression

**Fig 7:**
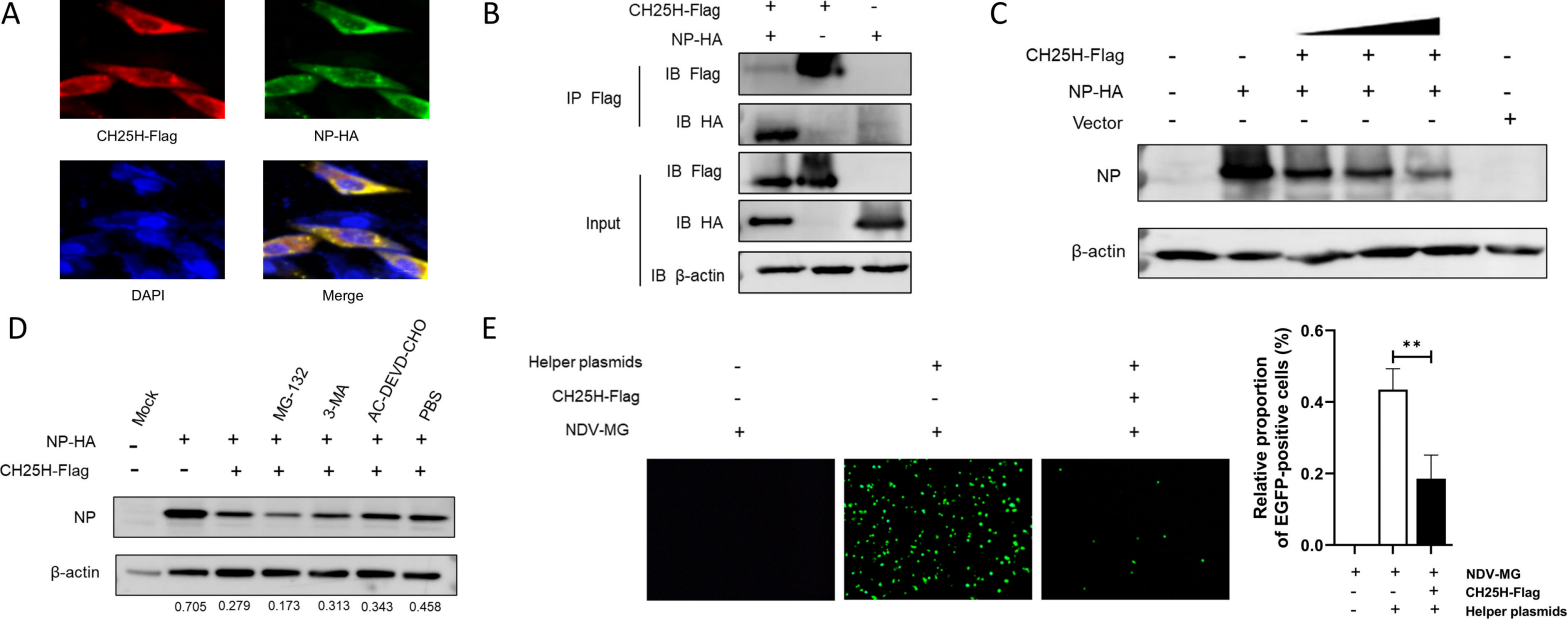
CH25H interacts with NDV NP and downregulates RNP activity. (**A**) DF-1 cells co-transfected with Flag-tagged CH25H and HA-tagged NP plasmids were fixed at 24 h post-transfection for immunofluorescence assays. A rabbit anti-Flag and mouse anti-HA antibodies were used as primary antibodies, followed by secondary antibodies conjugated with AF555 (anti-rabbit) and AF488 (anti-mouse). Nuclei were counterstained with DAPI. (**B**) DF-1 cells were co-transfected with recombinant plasmids encoding Flag-tagged CH25H and HA-tagged NP. At 24 h post-transfection, cell lysates were subjected to immunoprecipitation (IP) using an anti-Flag antibody. Whole-cell lysates and IP complexes were analyzed by western blot analysis with anti-Flag and anti-HA antibodies. (**C**) DF-1 were transfected with varying doses of CH25H and NP-expressing plasmids for 24 h. The replication of NDV was evaluated by Western blot analysis of NP expression. (**D**) DF-1 cells co-transfected with CH25H and NP-expressing plasmids were treated with specific inhibitors: MG132 (20 µM), 3-methyladenine (3-MA; 5 mM), or Ac-DEVD-CHO (25 µM) at 24 h post-transfection. After 12 h, cell lysates were analyzed by western blot analysis to detect the expression levels of NP, CH25H, and β-actin. (**E**) DF-1 cells were co-transfected with the NDV-MG minigenome reporter plasmid and helper plasmids encoding NP, P, and L proteins, along with either a CH25H expression plasmid or a control vector. At 48 hpost-transfection, EGFP-positive cells were imaged and quantified. Data shown are representative of three independent experiments.

In order to further elucidate the mechanism by which CH25H inhibits NDV infection independent of its enzymatic activity, we screened for interactions between CH25H and NDV-encoded proteins. Flag-tagged CH25H and HA-tagged NP expression plasmids were co-transfected into DF-1 cells, followed by double immunofluorescence staining. As shown in Fig. 7A, CH25H colocalized with NDV NP in the cytoplasm. To validate this interaction, a coimmunoprecipitation assay was performed, revealing a strong interaction between CH25H protein and NP (Fig. 7B). Western blot analysis further demonstrated that CH25H protein downregulated NP expression in a dose-dependent manner (Fig. 7C). In eukaryotic cells, protein degradation occurs primarily through the ubiquitin-proteasome system, the autophagy-lysosome pathway, and apoptosis-mediated mechanisms (18). To investigate whether CH25H-induced NP degradation is mediated by these pathways, DF-1 cells co-transfected with CH25H and NP-expressing plasmids were treated with specific inhibitors: MG132 (20 µM, proteasome inhibitor), 3-methyladenine (3-MA; 5 mM, autophagy inhibitor), and Ac-DEVD-CHO (25 µM, apoptosis inhibitor). None of these inhibitors impaired CH25H-mediated NP degradation (Fig. 7D), suggesting an alternative degradation mechanism.

NP is a critical component of the viral ribonucleoprotein (RNP) complex, which drives viral transcription, replication, and serves as the structural core of virions. Viral RNA synthesis relies on the RNP complex composed of NP, P, and L proteins. Since CH25H targets NP and inhibits NDV infection, we hypothesized that CH25H may disrupt viral RNP activity. To test this hypothesis, we utilized a minigenome system to reconstitute the viral RNP complex and evaluate its activity. Cells were co-transfected with a minigenome reporter plasmid (NDV-MG) and helper plasmids encoding NP, P, and L proteins, along with either a CH25H expression plasmid or a control vector. As shown in Fig. 7D and E, EGFP reporter gene expression, driven by the reconstituted RNP complex, was significantly suppressed in cells expressing CH25H, indicating that CH25H effectively inhibits RNP activity. In summary, these results demonstrate that CH25H interacts with NP and specifically interferes with viral RNP activity, thereby contributing to its antiviral mechanism.

## DISCUSSION

It is well known that the innate immune system, as the first line of defense against pathogenic infections, plays a crucial role in the host immune response to clear viruses. Recently, it has been reported that chicken CH25H is one of the ISGs induced by chicken interferon-alpha (IFN-α) in DF-1 cells. However, chicken CH25H restricts ALV-J infection in a manner dependent on enzyme activity product 25HC, rather than through type I and II IFNs (8). In this study, we found chicken CH25H expression was upregulated upon NDV infection. CH25H affects NDV replication through both enzyme activity-dependent and -independent mechanisms.

Lipid rafts are dynamic membrane microdomains characterized by the preferential enrichment of cholesterol, alongside sphingolipids and specific associated proteins (24). These microdomains play a critical role in various cellular processes, including viral infection (25, 26). The antiviral effect of 25HC has been demonstrated against a broad spectrum of viruses, both enveloped and non-enveloped, through its ability to regulate the lipid content and distribution within cell membranes. This regulation disrupts membrane-dependent steps in the viral replication cycle, including adsorption, entry, uncoating, biosynthesis, assembly, and release. In this study, we found that 25HC significantly inhibits the infection of NDV, a prototype of paramyxoviruses. Further mechanistic investigations revealed that the antiviral effect of 25HC against NDV is primarily mediated through its interference with viral internalization (Fig. 5). NDV entry into host cells is facilitated by two glycoproteins: hemagglutinin-neuraminidase and fusion (F) proteins. Cholesterol plays a pivotal role in the membrane fusion process between the viral envelope and the host cell membrane, a step initiated by viral fusion proteins. Our findings align with previous studies, such as Martín et al., which demonstrated that depleting cholesterol from target membranes impairs NDV entry into host cells (27). Similarly, for another paramyxovirus, Nipah virus, cholesterol levels have been shown to influence both an early F-triggering step and a late fusion pore formation step during the membrane fusion cascade (28). Together, these observations emphasize the critical role of cholesterol in paramyxovirus entry and underscore the potential of 25HC as a broad-spectrum antiviral agent targeting lipid-dependent stages of viral infection.

Recombinant viruses have proven to be invaluable tools for elucidating the intricate interactions between viruses and their hosts, particularly at the virus-cell interface. To investigate the impact of CH25H on NDV replication, we constructed a recombinant NDV expressing CH25H using a previously established virus rescue system. Our results showed that the recombinant rNDV-CH25H exhibited significantly reduced growth in both DF-1 cells and chicken embryos compared to the parental NDV NA-1 strain (Fig. 3). These findings demonstrate that CH25H effectively restricts NDV replication. Similarly, to investigate the role of receptor transporter protein 4 (RTP4) or IFN-induced protein with tetratricopeptide repeats (Ifits) in restricting RABV replication, recombinant RABVs expressing RTP4 or Ifits were constructed. The recombinant RABV expressing RTP4 displayed markedly lower viral titers in various neuronal cell types, highlighting RTP4’s potent antiviral activity (29). In contrast, recombinant RABVs expressing Ifits replicated with comparable efficiency to the parent virus, suggesting that Ifits may have limited effects on RABV replication (30).

Interestingly, we observed that a catalytic mutant of CH25H lacking hydroxylase activity (CH25H-M) retained anti-NDV activity, albeit at a reduced level compared to wild-type CH25H (Fig. 6). This finding indicates that the antiviral function of CH25H is not entirely dependent on its enzymatic activity, suggesting the involvement of non-enzymatic antiviral mechanisms. Previous studies have shown that CH25H and its mutants directly interact with viral proteins to inhibit replication. For instance, CH25H interacts with PRRSV nsp1α, promoting its degradation through the ubiquitin-proteasome pathway (23). It also binds to the HCV NS5A protein, preventing dimer formation (21); interacts with HBV HBx protein, inhibiting its nuclear translocation (22); and reduces the maturation of Lassa virus (LASV) G1 glycoprotein N-glycan, thereby decreasing the production of infectious LASV virions (31). Inspired by these findings, we investigated whether CH25H interacts with NDV proteins. Our results revealed that CH25H binds to the viral NP and significantly reduces its expression in a dose-dependent manner (Fig. 7). Interestingly, this reduction is independent of lysosomal, proteasomal, and caspase pathways. Given that NP is essential for the replication of the viral genome, this interaction likely represents a novel antiviral mechanism of CH25H.

NP is the most abundantly expressed protein encoded by the NDV genome (32). During viral replication, NP along with P and L proteins assembles into RNP complexes with viral RNA, forming the fundamental units required for genome replication and transcription (33). Host restriction factors have been shown to target viral NP to inhibit replication. For example, Myxovirus resistance protein A interacts specifically with NP of Dabie bandavirus, disrupting its interaction with RNA-dependent RNA polymerase (RdRp) and consequently inhibiting RNP activity (34). Similarly, the host restriction factor Moloney leukemia virus 10 protein binds to the N-terminal arm of the Severe fever with thrombocytopenia syndrome virus NP, preventing NP aggregation, NP-RNA binding, and NP-RdRp interactions, in turn restricting the viral replication and pathogenicity in *vitro* and in *vivo* (35). In our study, a comparable mechanism was observed: CH25H interacted with NDV NP and inhibited its RNP activity. This inhibitor significantly reduced viral transcription and replication, underscoring the critical role of host factors in restricting negative-sense RNA viruses. Given the similarities between NDV and other negative-sense RNA viruses, we hypothesize that CH25H may also target the N-terminal of NP to inhibit RNP activity. This hypothesis provides a foundation for future research, which will focus on characterizing the interaction between CH25H and NDV NP and elucidating its impact on RNP assembly and viral replication.

In conclusion, our study demonstrates that chicken CH25H is upregulated during NDV infection and inhibits viral entry through its enzymatic product, 25HC. Notably, a catalytic mutant of CH25H (CH25H-M), which lacks hydroxylase activity, retains significant antiviral activity. Additionally, we discovered that CH25H interacts with the viral NP, resulting in a marked reduction in NP expression and the suppression of RNP complex activity. These findings provide new insights into the multifaceted antiviral mechanisms of CH25H, highlighting both enzyme-dependent and -independent pathways. This study provides further evidence that CH25H is a promising target for developing novel antiviral strategies against NDV and related pathogens.

## MATERIALS AND METHODS

### Cells, virus, and antibodies

The NDV NA-1 strain (GenBank: DQ659677) used in this research was isolated from China and identified as velogenic genotype VII virus to both geese and chickens. A recombinant NDV expressing enhanced green fluorescent protein (rNDV-EGFP) was previously generated (36). DF-1 cells were cultured in Dulbecco’s modified Eagle’s medium (DMEM) (Gibco) containing 10% (vol/vol) fetal bovine serum (Gibco), at 37°C with 5% CO_2_ atmosphere. The mouse anti-chicken CH25H polyclonal antibody and rabbit anti-NDV NP polyclonal antibody were prepared from purified CH25H protein and NP, respectively.

### Construction of plasmids and plasmid transfection

The coding sequence of CH25H (GenBank: NM_001277354.1) was amplified by polymerase chain reaction (PCR) using a specific pair listed in Table 1. The CH25H fragment was then cloned into the pCAGGS vector to generate a Flag-tagged expression plasmid pCAGGS-Flag-CH25H. Site-directed mutagenesis was employed to convert the histidine codons at positions 242 and 243 of CH25H into glutamine codons, resulting in the mutated plasmid, CH25H-M. All plasmid constructs were confirmed by sequencing.

**TABLE 1:** Primers used for cloning and real-time qPCR analysis^*a*^

Primer name	Sequence (5′−3′)	Application
CH25H-F-1 (Gallus)	CGGAATTCGCCACCATGAACTGCAGCGTGCGG	Construction of pCAGGS-CH25H
CH25H-R-1 (Gallus)	GGGGTACCTTACTTATCGTCGTCATCCTTGTAATCACTAGG	
CH25H-F-2 (Gallus)	GCTGCCATCAATCCATTCCTCCTC	qRT-PCR detection of CH25H (Gallus)
CH25H-R-2 (Gallus)	TCCATACCAACCGAAAGGCACAAG	
NP-F	TGCTCAGACCCGCCCTAACG	qRT-PCR detection of NP (NDV)
NP-R	TCGCTAACAGCAATCCGAAGACAG	
GAPDH-F (Gallus)	CCTCTCTGGCAAAGTCCAAG	qRT-PCR detection of GAPDH (Gallus)
GAPDH-R (Gallus)	CATCTGCCCATTTGATGTTG	
CH25H-M-F	CACCGCATCATGATCTCCAACAACTGAAGTTCAAGTCAAAC	Construction of pCAGGS-CH25H-M
CH25H-M-R	GTTTGACTTGAACTTCAGTTGTTGGAGATCATGATGCGGTG	

^*a*^ The underlining shows the restriction enzymes.

For transfection, the X-tremeGENE HP DNA Transfection Reagent was mixed with plasmid DNA at a ratio of 4:1 and added to DF-1 cells. The cells were incubated at 37°C in a humidified incubator containing 5% CO_2_.

### siRNA assay

Three pairs of siRNAs targeting the chicken *ch25h* gene (siRNA1 [5′-GCACUAGACUGCCAAACUUTT-3′, 5′-AAGUUUGGCAGUCUAGUGCTT-3′]; siRNA2 [5′-GCUCUUACUACACAGUAUUTT-3′, 5′-AAUACUGUGUAGUAAGAGCTT-3′]; and siRNA3 [5′-GGAGGACCAUUCAGGAUAUTT-3′, 5′-AUAUCCUGAAUGGUCCUCCTT-3′]) were designed and synthesized. The DF-1 cells growing in 24-well plates were transfected with siRNAs or a non-specific NC using GP-transfect-Mate transfection reagent following the manufacturer’s instructions. At 48 h post-transfection (hpt), the levels of CH25H were tested by quantitative real-time PCR (qRT-PCR).

### qRT-PCR

Total RNA was extracted from DF-1 cells using an RNA Extraction Kit and reverse transcribed into cDNA with HiScript qRT SuperMix, following the manufacturer’s protocols. qRT-PCR was then performed using the qPCR SYBR Green Master Mix. The individual transcripts in each sample were analyzed three times. The qRT-PCR conditions were as follows: initial denaturation at 95°C for 30 s, followed by 40 cycles of 5 s at 95°C and 34 s at 60°C. Gene-specific primer sequences were designed by primer design software, as shown in Table 1. Relative mRNA expression levels were normalized to GAPDH. The data were analyzed using the ΔΔCt method.

### Western blot analysis

Cells were lysed using RIPA Lysis Buffer (Beyotime, China) and the supernatant was collected by centrifugation at 12,000 × *g* for 10 min. Target proteins were separated by SDS-PAGE and transferred to polyvinylidene difluoride (PVDF) membranes. The membrane was blocked with 5% skim milk and incubated with the indicated Flag or HA monoclonal antibodies. HRP-conjugated anti-rabbit or anti-mouse IgG antibodies were used as secondary antibodies. Protein detection was performed using an enhanced chemiluminescent substrate.

### Co-immunoprecipitation (IP)

After plasmid transfection, cell samples were collected and lysed in Western and IP lysis buffer. The cell lysate supernatant was incubated with a Flag monoclonal antibody overnight at 4°C for IP, followed by incubation with protein A + G agarose at 4°C for 3 h. The immunoprecipitated samples were then separated by SDS-PAGE and transferred to PVDF membranes (Millipore, USA). Western blot analysis was performed using the specified antibodies.

### Virus titration

The TCID_50_ assay was performed to assess virus titration and infectivity. On day 0, Vero cells were seeded in a 96-well plate at a density of 1 × 10^4^ cells per well, and 200 µL maintenance medium (DMEM/2% FBS) was added to each well. On day 1, the cells were inoculated with serially diluted viruses (10^−1^ to 10^−11^ fold) at 37°C. The cells were further cultured for 3–5 days. The cells demonstrating the expected cytopathic effect were observed daily, and the TCID_50_ value was calculated by the Reed–Muench method. Each assay was performed in triplicate.

### Immunofluorescence staining

DF-1 cells were fixed with 4% paraformaldehyde for 15 min and then permeabilized with 0.1% Triton X-100 in PBS supplemented with 2% bovine serum albumin for 30 min at room temperature. The cells were subsequently incubated with Flag monoclonal antibody at a dilution of 1:200 for 1 h at 37°C. After three washes with PBS, the cells were incubated with a fluorescein isothiocyanate-conjugated goat anti-Rabbit IgG (H + L) antibody (Beyotime, China) at a dilution of 1:200 for 1 h at 37°C. After washing, nuclei were stained with 4′,6-diamidino-2-phenylindole (DAPI) for 10 min at room temperature. Laser-scanning microscopy was performed using an inverted fluorescence microscope.

### Cell viability assay

DF-1 cells were seeded in 96-well plates and incubated with 25HC at the indicated concentrations (0, 0.25, 0.5, 1, 2, and 4 µM) for 24 h. A total of 10 µL of CCK-8 reagent was added into each well. After 1 h, the absorbance at 450 nm was measured after being gently mixed in a shaker for 1 min to ensure homogeneous distribution of color.

### Rescue of recombinant virus

The CH25H ORF with conserved gene end (GE) sequences and gene start (GS) sequence was PCR-amplified using the primer pair and inserted into the pCI-NA-1 between the P and M genes, resulting in pCI-NA-CH25H. The rescue of recombinant virus was performed as described previously (36). Briefly, 293T were grown in six-well plates overnight to 80% confluency, then co-transfected with a total of 4 µg DNA consisting of a mixture of pCI-NA-CH25H with the NP-, P-, L-helper plasmids at a ratio of 4:2:1:1 using the Lipofectamine 3000 (Invitrogen) according to the manufacturer’s instructions. Five days after transfection, the supernatant was harvested and injected into the allantoic cavities of 9-to 11-day-old embryonated SPF eggs. The hemagglutination (HA) test was conducted after 5 days of incubation. The recovered viruses were named rNDV-CH25H.

### Minigenome reporter assay

To construct the minigenome reporter plasmid, the T7-trailer and leader sequences were amplified from plasmid pCI-NA-eGFP (36) using primer pairs T7P-Trailer-F/T7P-Trailer-R and Leader-F/Leader-R (Table 2), respectively. The EGFP gene was amplified using primers EGFP-F and EGFP-R. The HDV 84-nt antigenome ribozyme (HdvRz) sequence, flanked by the T7 polymerase terminator (T7Ter), was amplified in a three-round PCR using HDV-F and three downstream primers (HDV-R1, HDV-R2, and HDV-R3). The T7-Trailer-EGFP-Leader-HdvRz-T7Ter fragment was then constructed via overlap extension PCR and cloned into the pcDNA3.1 vector at the *Nhe* I site using the In-Fusion HD Cloning strategy with primers T7P-Trailer-F and T7t-R. The resulting plasmid was confirmed by Sanger sequencing, designated as NDV-MG.

**TABLE 2:** Primers used for minigenome construction^*a*^

Primer name	Sequence (5′3′)
T7P-Trailer-F	TTAATACGACTCACTATAGGGACCAAACAGAGATTTGGTGAA
T7P-Trailer-R	GCTGTACAAGTAAAGGCAATCGTACGCCAATC
EGFP-F	ACGATTGCCTTTACTTGTACAGCTCGTCCATGCCG
EGFP-R	TGCCACCATGGTGAGCAAGGGCGAGGA
Leader-F	CCCTTGCTCACCATGGTGGCatcggtagaaggttccctc
Leader-R	GGTGGAGATGCCATGCCGACCCACCAAACAGAGAATCTGTG
HDV-F	TTTGGTGGGTCGGCATGGCATCTCCACC
HDV-R1	ATACCGAAGTATCTGTTATCTATGCTAGTTATTGCTCAGCGGCGCCCTCCCTTAGCCATCCGA
HDV-R2	ATAGCCGCGCGAACGCGGCTATCTGTTGAAAAAAAACAGATAACAGATACCGAAGTATCTGTTATCTA
HDV-R3	CCCTCGAGTCGATCCGGATATAGTTCCTCCTTTCAGCAAAAAAAACAGATAGCCGCGCGAACGCGGCTA
T7t-R	AAACGCTAGCCAGCTTGGGTCTTCGATCCGGATATAGTTCC

^*a*^ The T7 promoter sequences are underlined.

To measure viral polymerase activity, BSR cells were co-transfected with the NDV-MG plasmid, helper plasmids encoding NP, P, and L proteins at a ratio of 4:2:1:1 (2 µg/well), together with the plasmid expressing CH25H (1 µg/well) or control vector as indicated. The cells were then incubated at 37°C. Forty-eight hours after transfection, EGFP-positive cells were visualized under a fluorescence microscope, and the number of EGFP-expressing cells was quantified.

### Statistical analysis

All data are presented as the mean ± standard deviation (SD) and were analyzed using one-way ANOVA with GraphPad Prism 9.0 software (USA). A *P* value of less than 0.05 was considered significant for all comparisons. *P < 0.05, **P < 0.01, ***P < 0.001.

## Data Availability

All data supporting the findings of this study are included within the article. The original data can be provided upon request to the corresponding author Jianzhong Wang (wjzd2005@163.com).
